# Carprofen elicits pleiotropic mechanisms of bactericidal action with the potential to reverse antimicrobial drug resistance in tuberculosis

**DOI:** 10.1093/jac/dkaa307

**Published:** 2020-08-13

**Authors:** Arundhati Maitra, Dimitrios Evangelopoulos, Alina Chrzastek, Liam T Martin, Aidan Hanrath, Ellie Chapman, Helen C Hailes, Marc Lipman, Timothy D McHugh, Simon J Waddell, Sanjib Bhakta

**Affiliations:** d1 Mycobacteria Research Laboratory, Institute of Structural and Molecular Biology, Department of Biological Sciences, Birkbeck, University of London, Malet Street, London WC1E 7HX, UK; d2 UCL Centre for Clinical Microbiology, University College London, Royal Free Campus, Rowland Hill Street, London NW3 2PF, UK; d3 Department of Chemistry, University College London, 20 Gordon Street, London WC1H 0AJ, UK; d4 Royal Free London NHS Foundation Trust, UCL-TB and UCL Respiratory, University College London, London NW3 2QG, UK; d5 Department of Global Health and Infection, Brighton and Sussex Medical School, University of Sussex, Brighton BN1 9PX, UK

## Abstract

**Background:**

The rise of antimicrobial drug resistance in *Mycobacterium tuberculosis* coupled with the shortage of new antibiotics has elevated TB to a major global health priority. Repurposing drugs developed or used for other conditions has gained special attention in the current scenario of accelerated drug development for several global infectious diseases. In a similar effort, previous studies revealed that carprofen, a non-steroidal anti-inflammatory drug, selectively inhibited the growth of replicating, non-replicating and MDR clinical isolates of *M. tuberculosis*.

**Objectives:**

We aimed to reveal the whole-cell phenotypic and transcriptomic effects of carprofen in mycobacteria.

**Methods:**

Integrative molecular and microbiological approaches such as resazurin microtitre plate assay, high-throughput spot-culture growth inhibition assay, whole-cell efflux inhibition, biofilm inhibition and microarray analyses were performed. Analogues of carprofen were also synthesized and assessed for their antimycobacterial activity.

**Results:**

Carprofen was found to be a bactericidal drug that inhibited mycobacterial drug efflux mechanisms. It also restricted mycobacterial biofilm growth. Transcriptome profiling revealed that carprofen likely acts by targeting respiration through the disruption of membrane potential. The pleiotropic nature of carprofen’s anti-TB action may explain why spontaneous drug-resistant mutants could not be isolated in practice.

**Conclusions:**

This immunomodulatory drug and its chemical analogues have the potential to reverse TB antimicrobial drug resistance, offering a swift path to clinical trials of novel TB drug combinations.

## Introduction

In 2018, 10 million people developed active TB and an estimated 1.5 million died from the disease.^1^ Since the introduction of the multidrug therapeutic regimen for drug-susceptible (DS)-TB, research priorities have focused elsewhere, resulting in only three new anti-TB drugs being released over the last 40 years. Some *Mycobacterium tuberculosis* strains, in the meanwhile, have acquired genetic mutations that render them resistant to most antibiotics.^2^ As the number of effective antibiotics falls, drugs that can be rolled out immediately have fuelled interest in drug repurposing, the process of identifying commercially approved drugs used for other indications or diseases that also exhibit antimycobacterial properties.^3–7^ This approach benefits from prior knowledge of the drug’s safety and metabolic properties, as these are already approved by the FDA and EMA for use in humans.

Non-steroidal anti-inflammatory drugs (NSAIDs) are widely used for pain relief. The common NSAIDs aspirin and ibuprofen have been reported to decrease the size and number of lung lesions and the bacillary load and to improve survival rates in murine models of TB.^8^^,^^9^ Ibuprofen and diclofenac enhance the activity of pyrazinamide and streptomycin, respectively, when administered in the initial phase of TB treatment in mice.^10^^,^^11^ Antimycobacterial activity of oxyphenbutazone has been reported against replicating, non-replicating and drug-resistant forms of the pathogen while also working synergistically with several anti-TB drugs. An *in vitro* infection model indicated that diflunisal and bromfenac possess antimycobacterial properties.^12^ Conversely, a study demonstrated that ibuprofen and celecoxib increase bacterial loads in mice infected via aerosol challenge as opposed to the IV infection route adopted in the investigations discussed above.^13^ Another study (NCT02602509) using a whole-blood bactericidal activity model demonstrated that celecoxib did not potentiate rifampicin or pyrazinamide.^14^ This highlights the importance of developing and testing on appropriate animal/*ex vivo* models, as well as the gap in knowledge concerning at which stage of infection (early or late) NSAIDs should be administered for maximal benefit.

A limited number of clinical studies are currently exploring the potential of NSAIDs in TB control. NCT02060006 is a Phase 3 trial to identify whether meloxicam can prevent TB immune reconstitution inflammatory syndrome, a serious clinical issue in HIV-coinfected TB patients. Other trials include a pilot (Phase 2) clinical study (NCT02237365) of aspirin as an adjunctive treatment for TB meningitis and a trial (Phase 2) of ibuprofen for the treatment of XDR-TB in addition to the standard therapy (NCT02781909). The immunomodulatory function of NSAIDs (etoricoxib) in increasing the protection offered when administered alongside a TB vaccine is being investigated in the trial NCT02503839.

A previous drug-screening study from our group revealed carprofen, an NSAID, to selectively inhibit the growth of replicating, non-replicating and MDR clinical isolates of *M. tuberculosis* at 40 mg/L.^15^

Though the general antibacterial activity of NSAIDs has been reported independently for a few years,^4^^,^^16^ the mechanisms by which they exert their antibiotic action on *M. tuberculosis* are not understood.

In this study, we reveal carprofen’s potential to reverse antimicrobial resistance in TB through its pleiotropic modes of action. This, in addition to its role as an immunomodulator in the host, offers great potential in anti-TB therapy.

## Methods

All chemical reagents were sourced from Merck (previously Sigma–Aldrich) unless otherwise mentioned.

### Experimental models and subject details

*M. tuberculosis* H37Rv (ATCC 25618) and *Mycobacterium smegmatis* mc^2^155 (ATTC 700084) cultures were maintained at 37°C as standing cultures or with 150 rpm shaking, respectively, using Difco Middlebrook (M)7H10 agar/7H9 broth (BD Biosciences/Merck) supplemented with 0.2% (v/v) glycerol, 0.05% (v/v) Tween 80 (broth only) and 10% (v/v) Middlebrook OADC growth supplement (BD Biosciences/Merck) or Middlebrook ADC, respectively. All bacterial cultures were passaged at least twice from cryogenically preserved stocks before use in experiments.

### Resazurin microtitre plate assay (REMA)

Mid-log phase (OD_600_ ∼1.0–1.20) *M. smegmatis* was diluted (10^−3^) in M7H9 and 100 μL of inoculum was tested against a concentration range of carprofen. The plates were incubated at 37°C for 24 h, then 30 μL of resazurin solution (5 mL of resazurin reagent mixed with 5 mL of 20% Tween 80) was added to each well and incubated at 37°C for 6–9 h. The MIC was determined as the minimum drug concentration where the colour of the suspension in the microtitre plate well remained blue.

### Inhibition of multidrug efflux pumps in a whole-cell setting

#### Accumulation assay

Early-log phase bacteria (OD_600_ ∼ 0.8) were adjusted to OD_600_ = 0.4 and resuspended in 1× PBS. Each reaction contained 4–6 × 10^7^ cfu/mL in PBS for *M. smegmatis*, 0.4% glucose, 0.5 mg/L ethidium bromide (EtBr) and subMIC concentrations of the antimicrobial drugs being tested. Negative controls contained all components but with the bacterial suspension replaced with 1× PBS. The experiment was performed in 96-well plates that were placed in a fluorimeter (BioTek Synergy 2) programmed with the following parameters: wavelengths of 544 nm and 590 nm for excitation and detection of fluorescence, an incubation temperature of 37°C and a cycle of measurement every minute for a total period of 60 min.

#### Efflux assay

The cells were prepared as above and packed with EtBr by incubating with 1 mg/L EtBr and verapamil at 25°C for 1 h. Following incubation, cells were centrifuged at 4°C and the supernatant removed before addition of cold 1× PBS to the bacterial pellet to obtain a bacterial suspension. EtBr efflux was measured in a 96-well plate in 100 μL volumes containing subMIC concentrations of the compounds, 0.4% glucose and 4–6 × 10^7^ cfu/mL. Fluorescence was read using the parameters described above. All experiments were performed in triplicate.

### Growth and maintenance of mycobacterial biofilm

For cultivation of biofilms, *M. smegmatis* was grown to late-log phase (OD_600_ > 6.0), harvested and resuspended (1:100 dilution) in Sauton’s medium. The bacterial suspension was incubated at 37°C for 5 days without shaking.

### cfu counting and OD determination of biofilms

Biofilms were grown in polypropylene (PPE) tubes then vortexed to result in a uniform cell suspension. One millilitre of the suspension was used to read the OD_600_ corrected against a blank containing sterile medium. One hundred microlitres of the bacterial suspension was diluted (dilution series 10^−3^ to 10^−8^ of the original) and 100 μL of each dilution was spread on M7H10 plates. The plates were incubated for 3 days at 37°C before counting and SD was calculated.

### Confocal laser scanning microscopy

*M. smegmatis* biofilms were grown in 6-well plates (Corning), pre-loaded with sterilized glass coverslips. Sauton’s medium containing 0.25× MIC of carprofen was used for the experiments. Drug-free and solvent controls were also set up. Coverslips were removed, making sure they lifted the biofilm, and 20 μL of 6 μM propidium iodide (PI) and 20 μL of 4 μM Nile red were added onto each coverslip placed on a slide separately as the excitation and emission wavelengths for these dyes overlap. Each dye was added 45 min prior to visualizing with confocal laser scanning microscopy (Leica, SP5) to allow the dye to penetrate. Biofilms were analysed though a series of images in the *z*-axis, followed by selection of optical planes.

### Crystal violet staining of biofilms

Once biofilms were formed in 96-well plates, the medium containing planktonic cells was aspirated and the plate was dried. Crystal violet (0.1% w/v) was added to the biofilms and left for 30 min at room temperature. The stain was removed and 30% acetic acid was added to solubilize the biofilm. Absorbance was measured at OD_600_ using a plate reader (BioTek Synergy 2). All experiments were performed in three independent biological replicates.

### Quantification of extra-polymeric substance (EPS)

Biofilms were grown in PPE tubes in the presence of a dilution series of carprofen (2–0.25× MIC; 500–62.5 mg/L). Solvent and drug-free controls were also set up. The biofilm was disrupted by gentle vortexing and moved to 15 mL centrifuge tubes, followed by centrifugation at 3000 **g** for 1 h at 25°C. The supernatant and pellet were moved into separate microfuge tubes and filtered through 0.22 μm filters to remove whole cells and debris. Each fraction was analysed for protein and carbohydrate content using the Bradford Assay and the Phenol Sulphuric Acid Assay,^17^ respectively. Twenty-five microlitres of each fraction was added to a 96-well round-bottom black plate (Corning) followed by 15 μL of 4 μM Nile red and incubated for 15 min prior to reading at 488–530 nm (excitation) and 575–580 nm (emission) to estimate lipid content. All experiments were performed in three independent biological replicates.

### RNA extraction

Mid-log phase cultures of *M. tuberculosis* (10 mL) were treated with 10× MIC carprofen (400 mg/L), ibuprofen (750 mg/L), ketoprofen (750 mg/L) or isoniazid (5 mg/L) for 4 h. The cultures were harvested by addition of 4 volumes of GTC solution (5 M guanidine thiocyanate, 0.1 M Tris-HCl, pH 7.5, and 0.1 M β-mercaptoethanol). Total RNA was extracted using the FastRNA^®^ Blue Kit (MP Biomedicals), following the manufacturer’s instructions.^18^^,^^19^

The RNA was then further purified using the RNeasy kit (QIAGEN), following the manufacturer’s instructions. To remove genomic DNA contamination, the RNA samples were treated with the Turbo DNA-free Kit (Ambion), according to the manufacturer’s instructions. Spectrophotometry was performed (NanoDrop 2000 machine, Thermo Scientific) to quantify the RNA concentration.

### Microarray analysis

Mycobacterial RNA (2 μg) was directly labelled with Cy3 fluorophore using the Universal Linkage System (ULS, Kreatech Diagnostics). Microarray hybridizations from three biological replicates were conducted, as previously described,^20^^,^^21^ using an *M. tuberculosis* microarray (Agilent Technologies) designed by the Bacterial Microarray Group at St. George’s (ArrayExpress accession number ABUGS-41). Significantly differentially expressed genes were identified comparing drug-treated to carrier control (DMSO-treated) using a moderated *t*-test (*P* value <0.05 with Benjamini and Hochberg multiple testing correction) and a >3.5-fold change. Significant overlaps in transcriptional signatures were identified using hypergeometric probability. Fully annotated microarray data have been deposited in Array Express (accession number E-MTAB-6191).

### Membrane potential assay

The membrane potential assay was performed using the BacLight™ Bacterial Membrane Potential Kit (Thermo Fisher Scientific) with slight modifications. *M. smegmatis* culture (OD_600_ = 1) was resuspended in PBS. Two hundred microlitres of the cell suspension was added to the wells of a black, flat-bottom 96-well plate. To each well, 2 μL of the DioC_2_(3) (provided in the kit) and 2 μL of drug (or DMSO) were added. For isoniazid, ¼× MIC was used, whereas for carprofen and CCCP, various concentrations were tested. The plate was incubated in the dark at 37°C for 30 min. It was read in a plate reader (BioTek Synergy 2), with excitation at 485 nm and emission readings taken at 540 nm and 620 nm. The ratio of red/green fluorescence was calculated for each well and the values were normalized. All experiments were performed at least in triplicate (*n *=* *3), with the drug-free and isoniazid controls performed using *n *=* *6.

### High-throughput spot-culture growth inhibition assay (HT-SPOTi)

HT-SPOTi was used to assess the MIC of carprofen and 2-(6-chloro-9*H*-carbazol-3-yl)acetic acid against *M. smegmatis* as described in our earlier publications.^15^^,^^22^^,^^23^ The plates were incubated at 37°C for 2 days. The MIC was determined as the lowest concentration of the compound where visible mycobacterial growth was not observed.

## Results and discussion

### Carprofen inhibits efflux pump activity in mycobacteria

**Figure 1.**
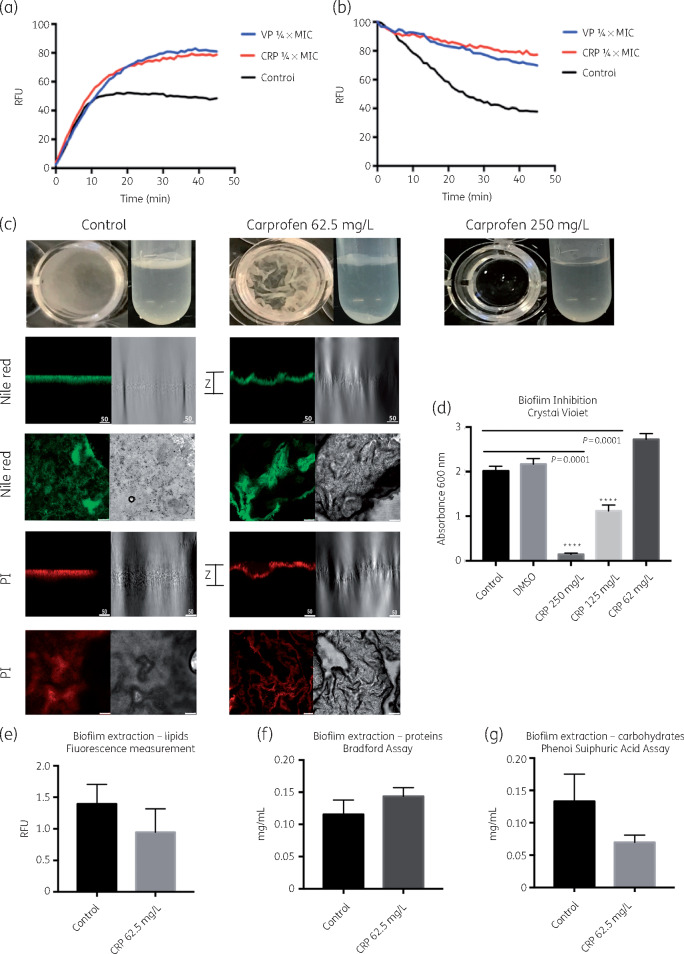
Whole-cell phenotypic effects of carprofen treatment on *M. smegmatis*. (a) Inhibition of efflux pumps in the presence of subMIC concentration of carprofen (CRP) (0.25× MIC, 62.5 mg/L) as seen through the accumulation of EtBr and resulting increase in the fluorescence readout. Verapamil (VP) (0.25× MIC, 50 mg/L) was used as positive efflux pump inhibitor control. Untreated cells served as the negative control. The experiments were performed in triplicate (*n *=* *3) and the graph was plotted using the averages. (b) Efflux of EtBr from pre-saturated cells in the presence of carprofen and verapamil, showing retention of the efflux pump substrate. (c) Complete inhibition of biofilm formation is observed in the presence of 250 mg/L (1× MIC) of carprofen in the PPE tubes and 96-well plates within a timescale of 5 days. Confocal images of stained extracellular DNA and lipids show distinct differences between carprofen-treated and untreated biofilms, especially their wrinkled surface, as captured in the *z*-axis images. (d) Concentration-dependent effect of carprofen on biofilm formation, as quantitated by crystal violet staining of the biofilms. Panels e, f and g show the amount of EPS, namely lipids, proteins and carbohydrates that make up the biofilms of the untreated control and carprofen-treated biofilms (0.25× MIC).

REMA assays determined the MIC of carprofen for *M. smegmatis* to be 250 mg/L. EtBr (a known substrate for bacterial efflux pumps) accumulation and efflux assays performed on *M. smegmatis* exposed to a subinhibitory concentration of carprofen (62.5 mg/L, 0.25× MIC), showed carprofen to inhibit efflux pumps to the same extent as the potent efflux pump inhibitor verapamil (Figure 1a and b).

Inhibition of drug efflux as part of a combination therapy can alter the efficacy of the treatment in three ways: (a) potentiate treatment (increase effectiveness at lower doses) in drug-susceptible bacilli; (b) reverse resistance (partially or completely) in some genetically resistant bacilli; and (c) shorten the duration of treatment by targeting ‘persister’ or ‘drug-tolerant’ phenotypes. These routes of tackling antimicrobial resistance offer great promise.^24^^,^^25^ Verapamil is a Ca^2+^ channel blocker used to treat cardiovascular disorders and has been shown to restore the susceptibility of MDR-TB strains to rifampicin, isoniazid and bedaquiline, among others.^26^ A similar investigation on carprofen is required. Whether carprofen inhibits efflux via direct binding to the pumps or through indirect mechanisms such as ATP sequestration or disturbing the membrane potential, or a combination of these strategies, is still to be determined. An additional gap in our understanding is whether carprofen can serve as a substrate for the pumps.

In bacteria, verapamil is believed to target the ATP-dependent multidrug transporters and MDR pumps by blocking the generation of the proton motive force^26^ and this could be a likely mechanism of action for carprofen. One of the molecular targets of carprofen in humans is cyclooxygenase-2 (COX-2), an enzyme that has been under investigation for its role in regulating efflux mechanisms via MDR1 proteins and development of a drug-resistant phenotype in cancerous tissue.^27^ Celecoxib is a COX-2 selective inhibitor that has been shown to reverse the drug resistance of cancers and bacteria alike.^28^^,^^29^ We expect both drugs to share some commonalities in their mechanism of action; however, unlike celecoxib, carprofen directly kills mycobacteria.

### Carprofen affects mycobacterial biofilm phenotype

Bacterial efflux pumps are implicated in quorum sensing (QS), which plays a crucial role in the regulatory processes of attachment and dispersion of biofilms^30^^,^^31^ mediated via small intracellular signalling molecules. Salicylic acid and propionic acid moieties and their derivatives are common structural features in NSAIDs that have been previously reported to inhibit biofilm formation or to disrupt the mature biofilms of many bacterial and fungal species such as *Candida* spp.^32^ Therefore, we developed a mycobacterial biofilm model to test the effect of carprofen on biofilm formation. At its effective MIC against planktonic *M. smegmatis* cells (250 mg/L), carprofen inhibited biofilm formation (Figure 1c), as was also noted by OD measurements (Figure 1d). Treatment with 0.25× MIC carprofen did not have any significant reduction on the viability of the biofilm (Figure S1, available as Supplementary data at *JAC* Online). Therefore, 0.25× MIC was used to measure the effect of carprofen on the EPS of mycobacterial biofilms. At this concentration, carprofen affected the levels of carbohydrates, but not proteins or lipids, of the mycobacterial biofilms (Figure 1e, f and g). These results need further investigation in the light of the transcriptomic signature of carprofen exposure as described below. Further experiments to understand whether carprofen can break down previously formed biofilms and the effect of drug exposure on the carbohydrate metabolites within the cell need to be performed.

Confocal microscopy using PI and Nile red, dyes that specifically stain extracellular DNA and lipids, respectively, was used to determine whether carprofen affected these key biofilm constituents. Carprofen exposure resulted in distinctive changes to the patterns of aggregation of these macromolecules in mycobacterial biofilms (Figure 1c). Extracellular DNA is known to play a role in the architectural composition of biofilms and any modification in its release and aggregation may explain the phenotype observed in treated biofilms (Figure 1c). The *z*-axis cross-section of the biofilms reveals that carprofen-treated biofilms acquire a highly distinctive, wrinkled appearance. This was also observed when biofilms were grown in tubes or plates, whereas the control biofilms were smooth in appearance. Whether the changes in the appearance of the biofilm are due to morphological changes in the cell or purely due to differences in the extracellular DNA/lipid content and localization needs to be confirmed via other microscopic techniques.

### Carprofen disrupts membrane potential in M. tuberculosis

**Figure 2.**
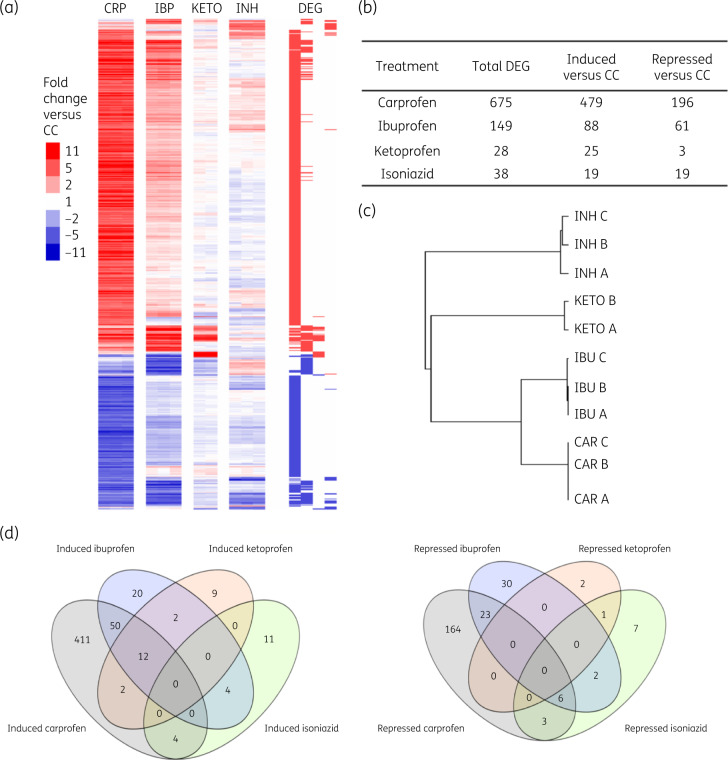
Transcriptional response of *M. tuberculosis* H37Rv to 4 h carprofen exposure (10× MIC, 400 mg/L). (a) Heatmap showing the NSAID drug signatures. Each column represents a drug treatment biological replicate and each row represents the expression profile of a gene relative to carrier control. Red colouring indicates induction and blue indicates repression. CRP, carprofen; IBP, ibuprofen; KETO, ketoprofen; INH, isoniazid; CC, carrier control. The genes identified as significantly differentially expressed in each drug comparison are marked as DEG. (b) Total number of genes that were significantly up- and down-regulated by drug exposure. (c) Hierarchical clustering of *M. tuberculosis* responses to drug exposure, showing similarity between replicate treatments (carprofen represented as CAR here). (d) The number of genes significantly modified by each drug exposure in comparison with carrier control. Overlap of genes induced or repressed by exposure to NSAIDs, showing minimal response to ketoprofen and discrete isoniazid signature.

To identify the endogenous target of carprofen, attempts were made to generate spontaneous drug-resistant mutant strains of *M. tuberculosis*; however, these were unsuccessful. A similar outcome was reported by Gold *et al.*^33^ while trying to generate strains resistant to another NSAID, oxyphenbutazone. The inability to generate spontaneous mutations that confer resistance to carprofen, although inconclusive, suggests that the drug may have pleiotropic mechanisms of action or targets a single physiological process at multiple points. This hypothesis was supported by the global impact of carprofen on *M. tuberculosis* gene expression (Figure 2a and Table S1). Carprofen modified the expression of 675 genes, in contrast to isoniazid, which affects the expression of <40 genes (Figure 2b).^34^

The carprofen mRNA signature was contrasted to DMSO-treated (carrier control) *M. tuberculosis* alongside other structurally similar but less anti-TB NSAIDs, ibuprofen and ketoprofen (Figure 2c and d). Isoniazid was included as a first-line cell wall-targeting comparator drug. Only 28 genes were significantly modulated by ketoprofen, an NSAID with no antimycobacterial property.

The *M. tuberculosis* response to carprofen did not overlap with drug signatures generated by first-line anti-TB drugs. None of the primary drug targets for isoniazid, rifampicin and pyrazinamide was modulated by carprofen treatment; those for ethambutol (*embAB*) were down-regulated. Overall, the distinct response to drug exposure shows that the mechanisms of action of carprofen are not the same as first-line anti-TB drugs currently in use, reducing the likelihood of cross-resistance.

The transcriptional response of *M. tuberculosis* to carprofen exposure was used to define the class of drug action. The 479 genes that were significantly induced by carprofen exposure were enriched with genes involved in the response to microaerophilic/anaerobic conditions, including enduring hypoxic response^35^ [hypergeometric probability (HGp) 3.43 × 10^−15^], the Wayne model of non-replicating persistence NRP-2^36^ (HGp 3.73 × 10^−9^) and the low potassium model of persistence^37^ (HGp 1.75 × 10^−30^). The functional category I.B.7, encompassing miscellaneous oxidoreductases and oxygenases, was also enriched (HGp 2.80 × 10^−8^), as were gene ontology (GO) terms for electron transporter activity GO:0005489/GO:0006118 (HGp 7.92 × 10^−6^). The drugs that resulted in the most similar signature to carprofen were valinomycin (HGp 1.63 × 10^−17^), pyrazinamide (HGp 3.82 × 10^−14^), pretomanid (HGp 1.38 × 10^−12^) and CCCP (HGp 3.31 × 10^−6^). A modular analysis of drug-responsive gene clusters, defined by Boshoff *et al.*,^38^ which significantly overlapped with the carprofen signature (HGp <0.004, induced GC13/87/121/58/141/101/81/132/60 and repressed GC25/36/95/71/26/70/8/1), mapped to agents (besides nitric oxide) that inhibit respiration. Valinomycin and CCCP were also the closest matches to carprofen’s mechanism of action using this independent unsupervised analysis.

Both valinomycin and CCCP are strong uncouplers that disrupt membrane potential.^39^^,^^40^ Shared transcriptomic signatures between these drugs and carprofen likely indicate imbalance in the respiratory chain or membrane potential that leads to cell death. Preliminary investigation indicates that carprofen can interfere with the membrane potential (Figure S2). However, the effect of carprofen on the membrane potential is much lower than that of CCCP.

The overlapping transcriptional profiles between pretomanid and carprofen may be a result of these compounds sharing a similar mode of action (Figure S3). Pretomanid is known to affect cell-wall metabolism as well as exert nitric oxide stress^41^^,^^42^ and the effect of carprofen on these mechanisms need to be investigated.

**Figure 3.**
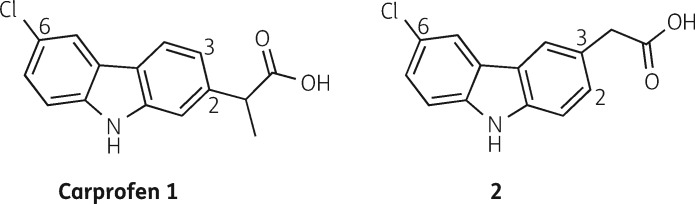
Chemical structure of carprofen (1) and 2-(6-chloro-9*H*-carbazol-3-yl)acetic acid (2).

### Novel carprofen analogue displays similar antimycobacterial effect to carprofen

In a study focused on the synthesis and evaluation of carprofen analogues, we have identified 2-(6-chloro-9*H*-carbazol-3-yl)acetic acid (structure 2 in Figure 3) as a carbazole with similar antimycobacterial properties (MIC_99_ of 250 mg/L for *M. smegmatis* mc^2^155) to carprofen [structure 1 in Figure 3]. This analogue lacks the α-methyl group of the propionic acid moiety at the 2-position of the carbazole ring in carprofen, which is required for the anti-inflammatory activity, and instead, 2-(6-chloro-9*H*-carbazol-3-yl)acetic acid has an ethanoic acid moiety at the 3-position. These structural alterations allow for a simple five-step chemical synthesis, utilising a Buchwald–Hartwig amination,^43–45^ followed by a modified Ackermark–Knölker cyclization^46^ as key steps in the assembly of the carbazole scaffold (Scheme S1 in ‘Synthesis Supplementary Information’ in the Supplementary data).

The identification of a carprofen analogue with similar antimycobacterial activity and a convenient synthesis route potentiates the evaluation of a diverse range of analogues. Further studies to explore the potential of 2-(6-chloro-9*H*-carbazol-3-yl)acetic acid and other carprofen analogues are currently ongoing within our research groups.

### Conclusions

Carprofen was developed by Roche^47^ and is commercially available as Rimadyl. The requirement for expensive starting materials, stringent reaction conditions and the lengthy synthetic steps made the development of other NSAIDs more attractive and the use of carprofen in humans stopped shortly after its release. A patent on a simplified process to synthesize carprofen and its derivatives has been filed in recent years.^48^ The identification of the carprofen analogue 2-(6-chloro-9*H*-carbazol-3-yl)acetic acid, which shows similar potency to carprofen in preliminary screening, may help to mitigate issues with the complexity of the chemical synthesis of carprofen, while retaining the desired antimycobacterial activity.

The anti-inflammatory activity of carprofen has been attributed to inhibition of phagocytosis by neutrophils.^49^ This property of carprofen could have far-reaching implications in the clinical treatment of TB as the primary route of pathogenesis of *M. tuberculosis* necessitates the initial, phagocytic uptake of bacteria by host cells.^50^

The pleiotropic effects of carprofen on mycobacteria and the possible likelihood of targeting membrane potential, efflux and mycobacterial biofilm formation limit the possibility for an emergence of spontaneously resistant mutants and offer alternative mechanisms of action to the current first-line anti-TB drugs. We also present a novel carprofen analogue, 2-(6-chloro-9*H*-carbazol-3-yl)acetic acid, with similar antimycobacterial activity to carprofen, which may be the first in a series of novel antimycobacterial carbazoles. Whilst acknowledging that our data relate to a mouse model, we believe that they warrant further exploration of the synergistic and immunomodulatory effects of this repurposed drug, with the ultimate goal of human studies.

## Supplementary Material

dkaa307_supplementary_data
