# Supplementary material for: Carprofen elicits pleiotropic mechanisms of bactericidal action with the potential to reverse antimicrobial drug resistance in tuberculosis
Source: J Antimicrob Chemother. 2020 Aug 13;75(11):3194–201. doi: 10.1093/jac/dkaa307 (PMC7566368; doi:10.1093/jac/dkaa307)

**Supplementary data**

**Figure S1:** Cfu count for biofilms treated with carprofen. Biofilms exposed to 0.25x MIC of carprofen were found to retain comparable viability as compared to the untreated control (n=3).

**
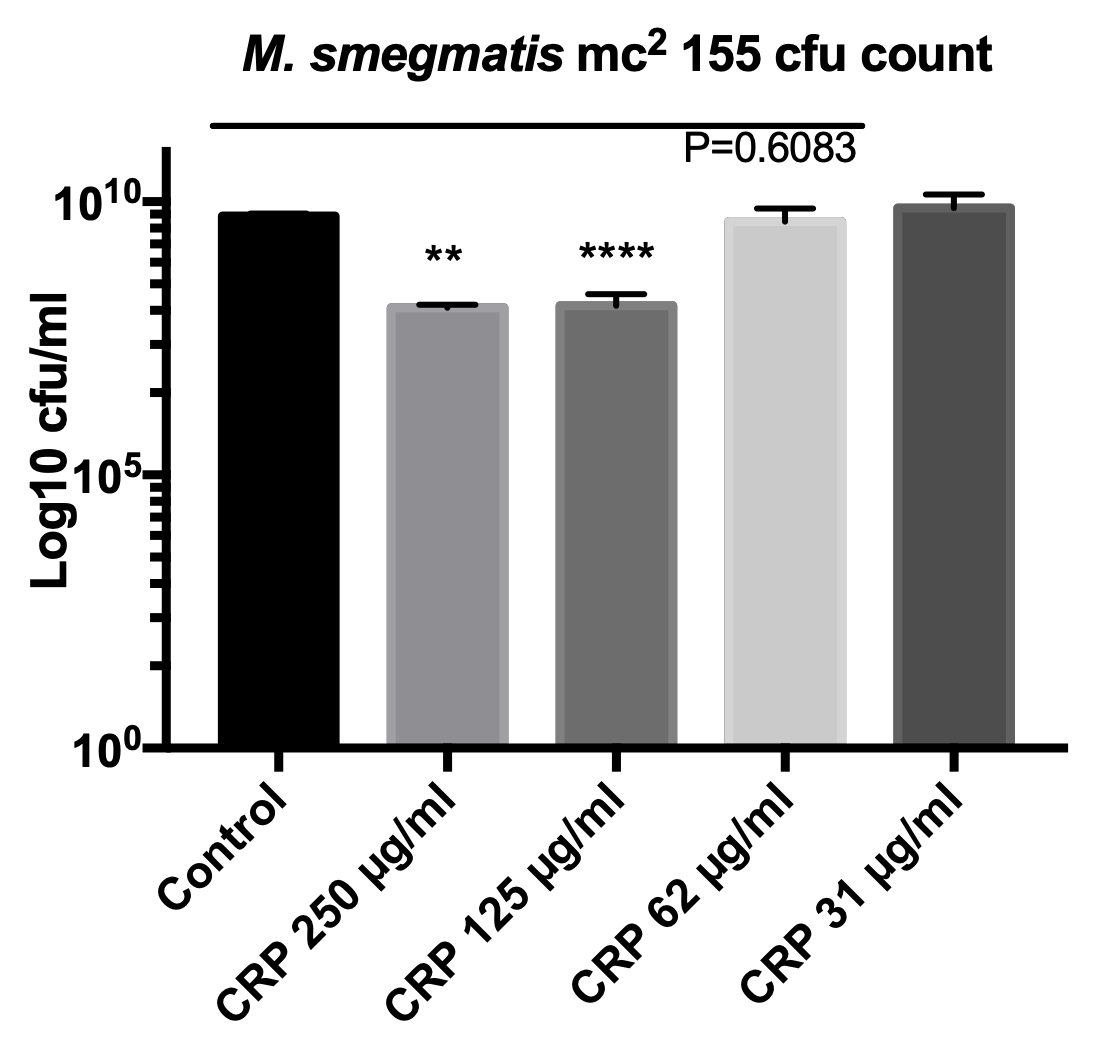
**

**Figure S2:** Membrane potential assay showing the potential of untreated cells (ratio normalised to 1) versus cells treated with isoniazid (INH) serving as a negative control, different concentrations of CCCP (tested from 1/4 - 1/16 x MIC) serving as a positive control and different concentrations of carprofen (CRP; tested from 1/4 - 1/16 x MIC).

**
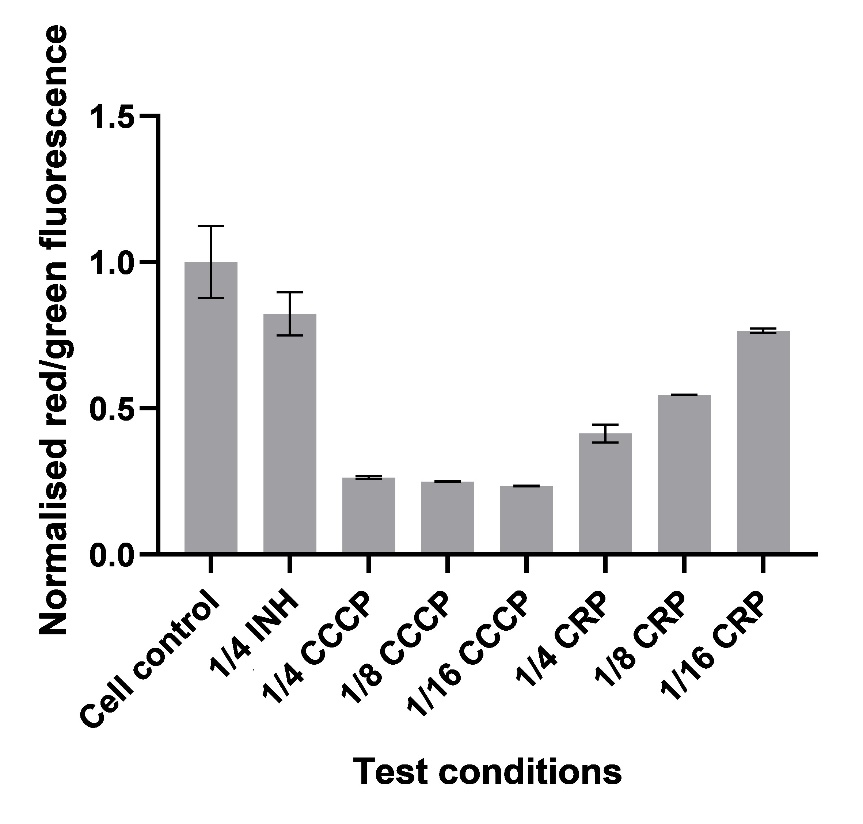
**

**Figure S3:** Chemical structures of pretomanid and carprofen **1**.


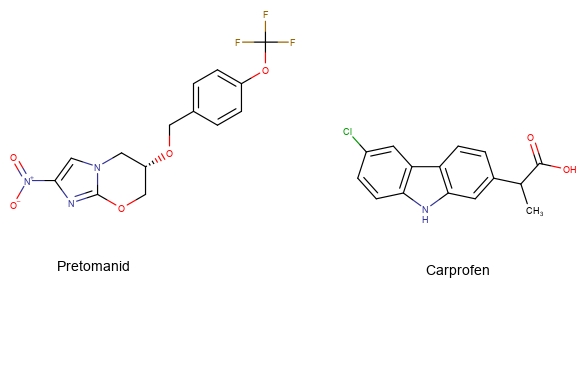


##### **Synthesis Supplementary Information**

|  |
| --- |
| **Scheme S1. Synthesis of 2** (a) *p*-toluenesulfonic acid, MeOH, reflux, 20 h, 91%; (b) Pd/C, H_2,_ MeOH, RT, 20 h, 89%; (c) 1-chloro-4-iodobenzene, Pd(OAc)_2_ (5 mol%), XPhos (10 mol%), Cs_2_CO_3_, toluene, reflux, 48 h, 57%; (d) Pd(OAc)_2_, (20 mol%), K_2_CO_3_, pivalic acid, 100 ^o^C, 18 h, 36%; (e) NaOH, CH_2_Cl_2_/MeOH, RT, 20 h, 32%. |

##### **Methyl 2-(4-nitrophenyl)acetate**^45^

##

To a solution of 4-nitrophenylacetic acid (1.50 g, 8.28 mmol) in methanol (20 mL), was added *para*-toluenesulfonic acid monohydrate (150 mg, 0.79 mmol) and the reaction mixture was heated at reflux for 20 h. The reaction was cooled to room temperature (RT) and the solvent was removed under reduced pressure. The crude product was taken up in ethyl acetate (20 mL) and washed with saturated NaHCO_3(aq)_ (3 x 20 mL). The organic layer was dried (sodium sulfate) and the solvent was removed under reduced pressure to afford the product^45^ as a white solid (1.47 g, 91%). M.p 57–59 ^o^C (ethyl acetate) (Lit. 50-57 ^o^C^45^); ^1^H NMR (700 MHz; CDCl_3_) δ 8.19 (2H, d, *J* = 8.7 Hz, 2 x 3-**H**), 7.46 (2H, d, *J* = 8.7 Hz, 2 x 2*-***H**), 3.74 (2H, s, C**H**_2_), 3.72 (3H, s, OC**H**_3_); ^13^C NMR (176 MHz; CDCl_3_) δ 170.7, 147.4, 141.4, 130.4, 123.9, 52.5, 40.9; *m/z* [ES+] 166 ([MH]^+^, 100%).


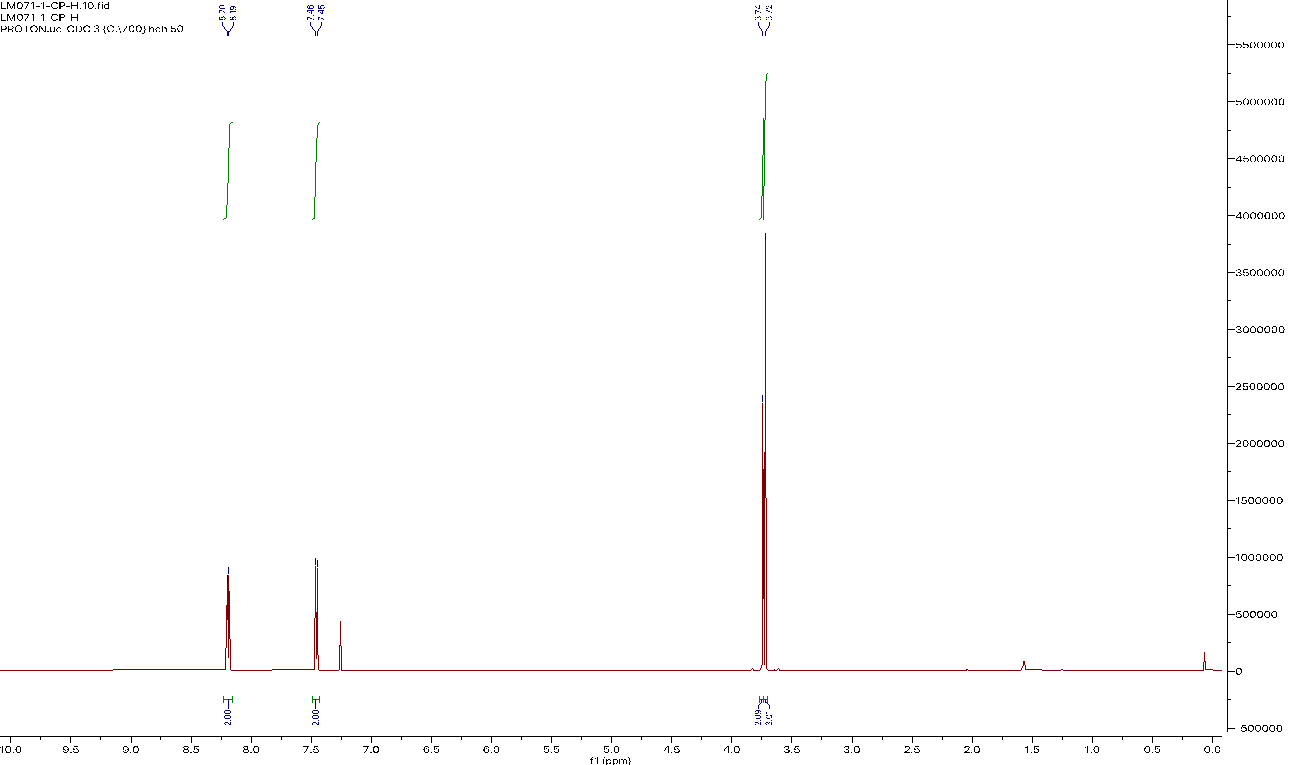


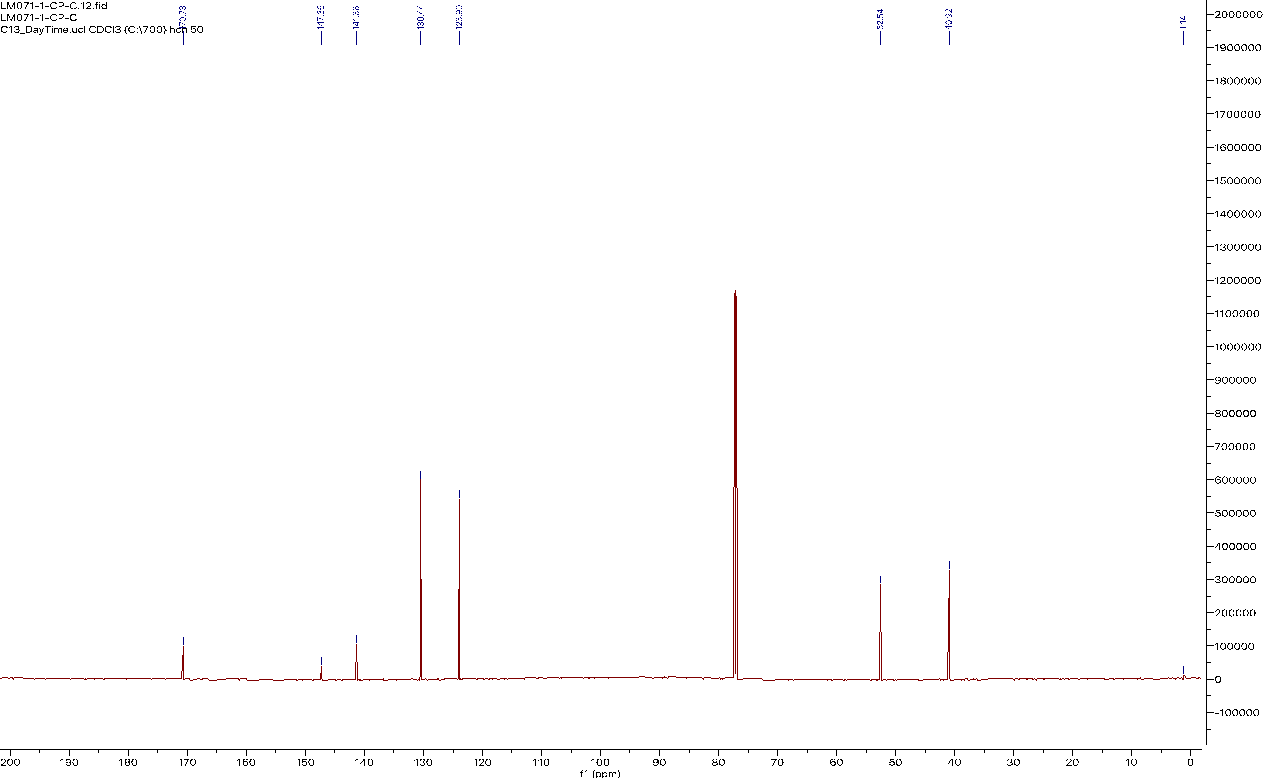


##### **Methyl 2-(4-aminophenyl)acetate**^46^

To methyl 2-(4-nitrophenyl)acetate (1.00 g, 5.12 mmol) in methanol (15 mL), Pd/C (10 wt%, 100 mg) was added. The vessel was flushed with argon (x 3), then placed under a hydrogen atmosphere and stirred for 20 h at RT. The reaction mixture was filtered through Celite, washed with methanol and the filtrate evaporated *in vacuo*. The crude product was added to water and the pH adjusted to 2 with HCl_(aq)_ (1 M). The aqueous layer was washed with ethyl acetate (3 x 20 mL), the pH of the aqueous layer was adjusted to 8 by the addition of NaHCO_3_ and extracted with ethyl acetate (3 x 20 mL). The combined organic layers were dried (Na_2_SO_4_) and the solvent removed under reduced pressure to afford the product^46^ as a pale-yellow oil (750 mg, 89%). ^1^H NMR (700 MHz; CDCl_3_) δ 7.06 (2H, d, *J* = 8.4 Hz, 2 x 2-**H**), 6.65 (1H, d, *J* = 8.4 Hz, 2 x 3-**H**), 3.67 (3H, s, OC**H**_3_), 3.51 (2H, s, C**H**_2_); ^13^C NMR (176 MHz; CDCl_3_) δ 172.6, 145.2, 130.1, 124.0, 115.4, 51.9, 40.3; *m/z* [ES+] 166 ([MH]^+^, 100%)


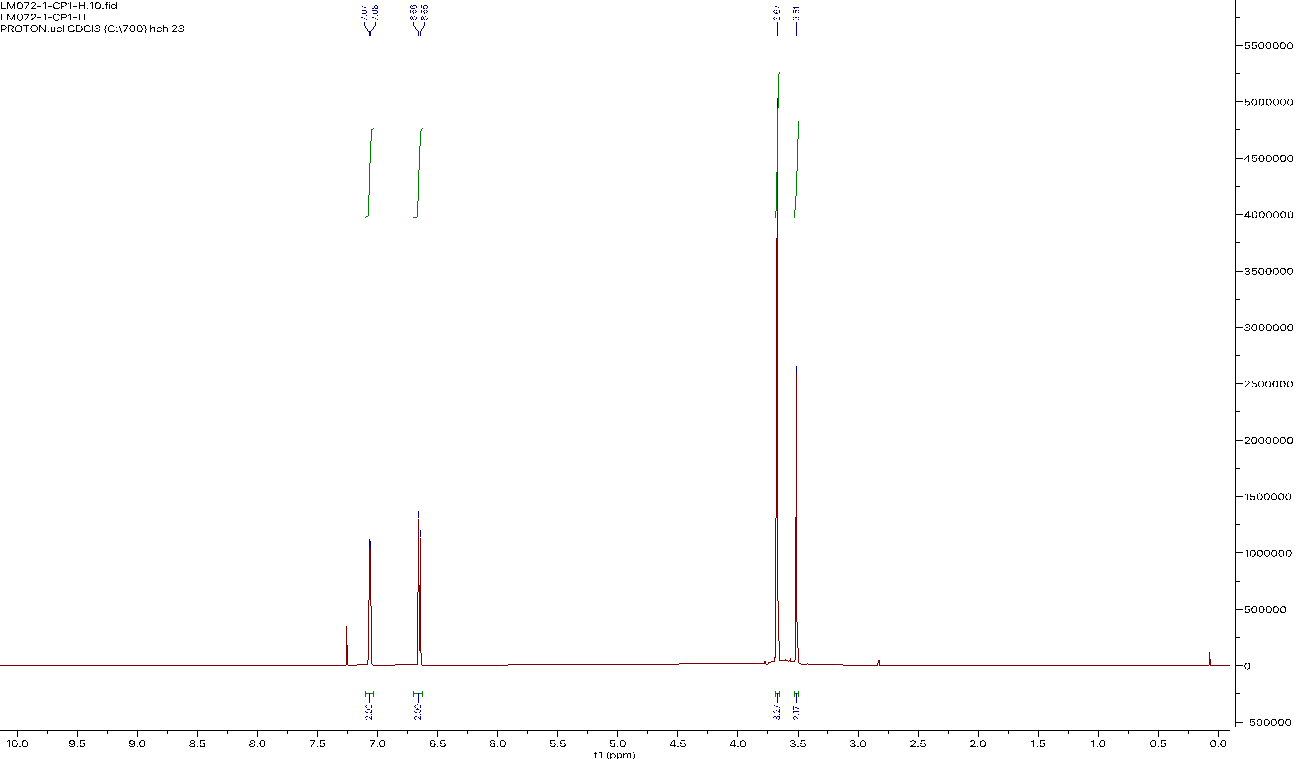


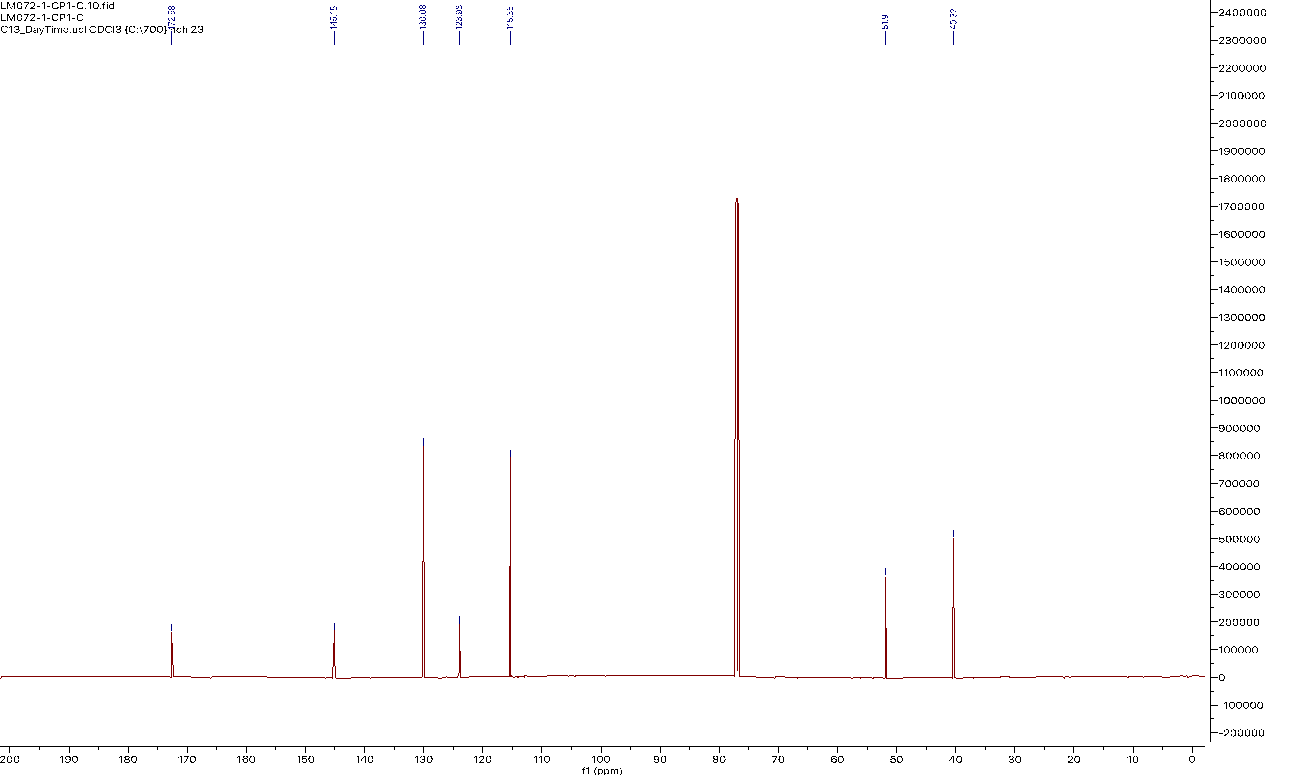


##### **Methyl 2-(4-[(4-chlorophenyl)amino)]phenyl)acetate**

Methyl 2-(4-aminophenyl)acetate (300 mg, 1.82 mmol), 1-chloro-4-iodobenzene (541 mg, 2.27 mmol), palladium acetate (20 mg, 5 mol%), XPhos (86 mg, 10 mol%) and caesium carbonate (858 mg, 2.63 mmol) were added to anhydrous toluene (10 mL) under an argon atmosphere. The reaction mixture was heated at reflux for 48 h. The solvent was removed under reduced pressure and the crude product purified by flash silica column chromatography (5-30% EtOAc in petroleum ether 40-60) to afford the product as a pale yellow solid (286 mg, 57%). M.p 94–96 ^o^C (ethyl acetate/pet. ether); ^1^H NMR (700 MHz; CDCl_3_) δ 7.20 (2H, d, *J* = 8.8 Hz, 2 x 3'-**H**), 7.18 (2H, d, *J* = 8.5 Hz, 2 x 2-**H**), 7.00 (2H, d, *J* = 8.5 Hz, 2 x 3-**H**), 6.97 (2H, d, *J* = 8.8 Hz, 2 x 2'-**H**), 5.66 (1H, s, N**H**), 3.70 (3H, s, OC**H**_3_), 3.57 (2H, s, C**H**_2_); ^13^C NMR (176 MHz; CDCl_3_) δ 172.5, 142.0, 141.8, 130.4, 129.4, 127.1, 125.7, 118.9, 118.4, 52.2, 40.6; *m/z* [ES+] 278.5 ([^37^Cl][MH]^+^, 35%), 276.5 ([^35^Cl] [MH]^+^, 100%).


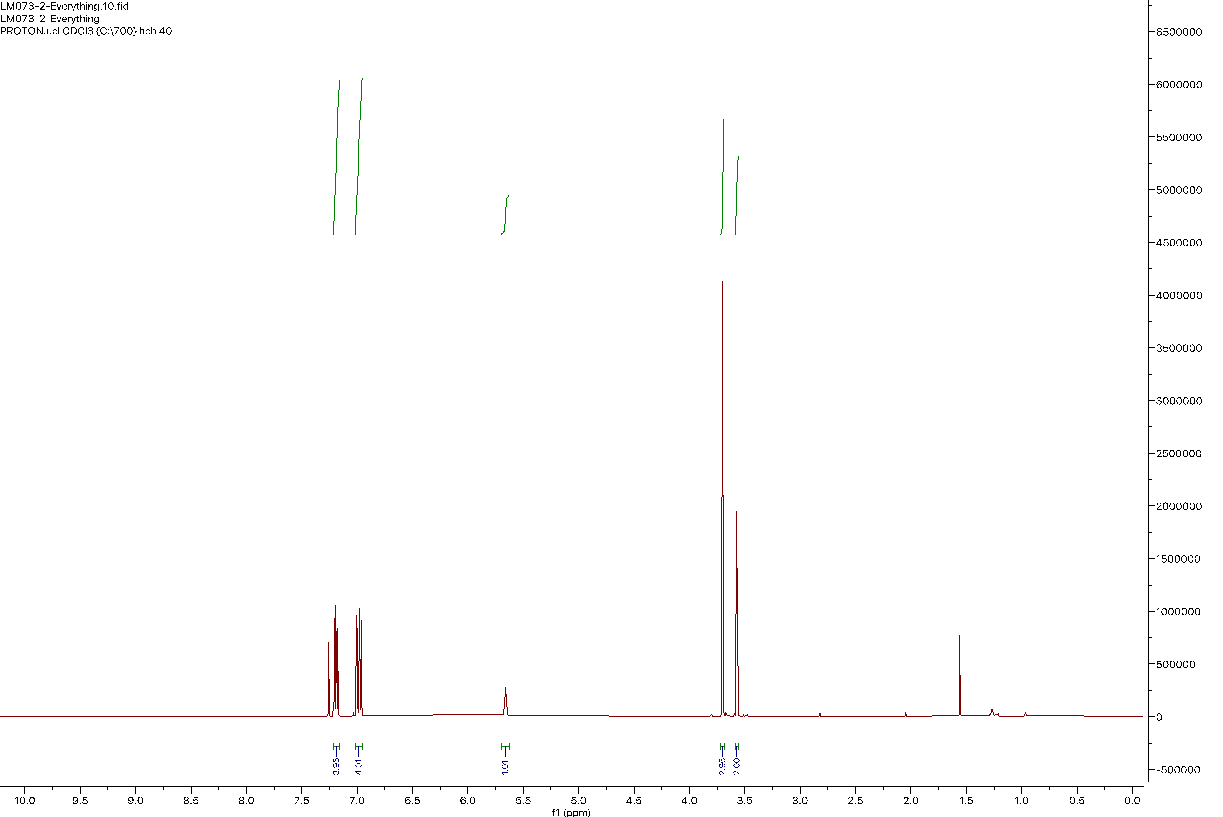


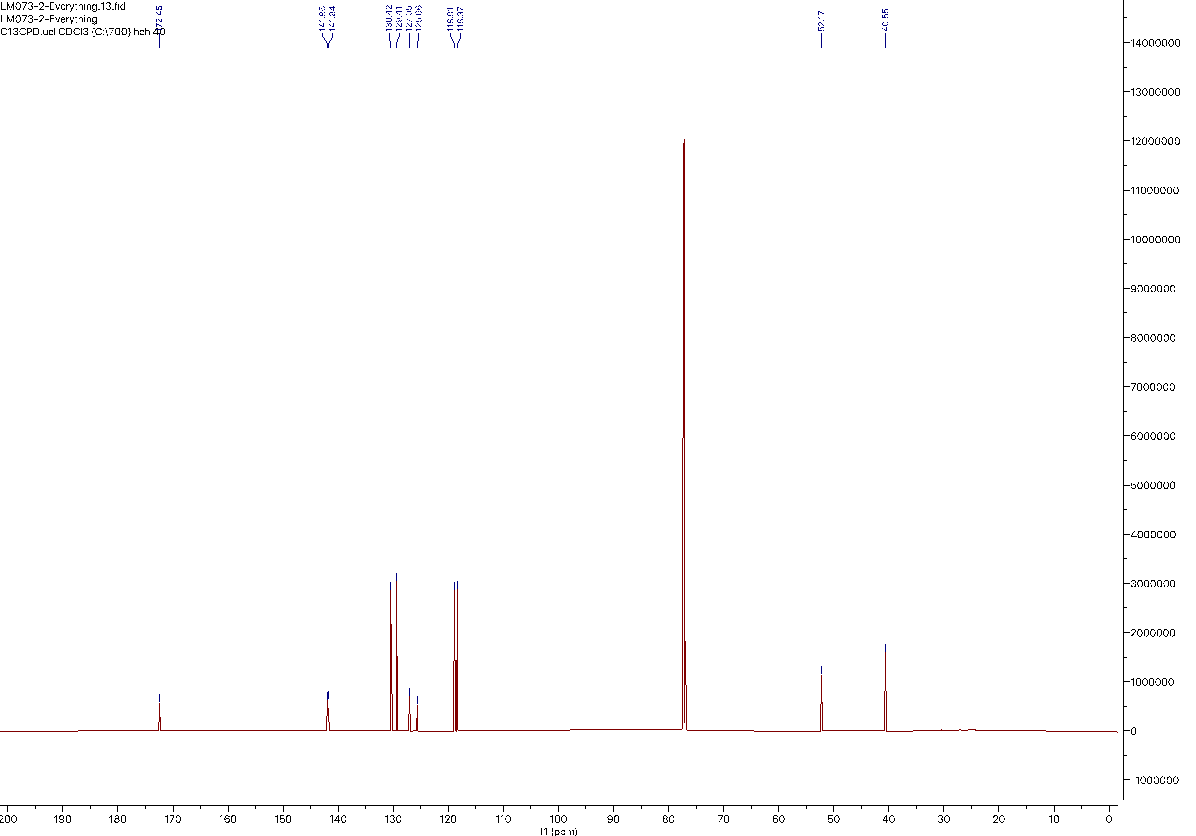


##### **Methyl 2-(6-chloro-9*H*-carbazol-3-yl)acetate**

Methyl 2-(4-((4-chlorophenyl)amino)phenyl)acetate (170 mg, 0.617 mmol), palladium acetate (28 mg, 0.12 mmol), potassium carbonate (17 mg, 0.12 mmol) and pivalic acid (1.00 g 10.5 mmol) were heated at 100 ^o^C for 24 h. The reaction was cooled to RT, filtered through Celite and washed with methanol. The filtrate was evaporated under reduced pressure. The crude product was added to diethyl ether (20 ml) and the organic phase washed with saturated NaHCO_3(aq)_ (3 x 20 mL). The organic phase was evaporated under reduced pressure and purified by flash silica column chromatography (5-30% EtOAc in petroleum ether 40-60) to afford the product as a yellow solid (61 mg, 36%). M.p 139–143 ^o^C (diethyl ether/pentane); *ν*_max_/cm^-1^ (neat) 3347, 1717; ^1^H NMR (700 MHz; CDCl_3_) δ 8.07 (1H, br. s, N**H**), 7.99 (1H, s, 5-**H**), 7.91 (1H, s, 4-**H**), 7.37–7.31 (4H, m, 1-**H**, 2-**H**, 7-**H**, 8-**H**), 3.79 (2H, s, C**H**_2_), 3.72 (3H, s, OC**H**_3_); ^13^C NMR (176 MHz; CDCl_3_) δ 172.8, 139.9, 138.2, 128.0, 126.2, 125.5, 125.1, 124.4, 122.9, 121.3, 120.3, 111.7, 111.0, 52.2, 41.3; *m/z* [ES+] 274 ([MH]^+^, 7%), 216 ([^37^Cl][M– COOMe]^+^, 30%], 214 ([^35^Cl][M–COOMe]^+^, 100%); *m/z* [HRMS, ES+] found [M]^+^ 273.0561. C_14_H_10_^35^ClNO_2_ requires 273.0551.


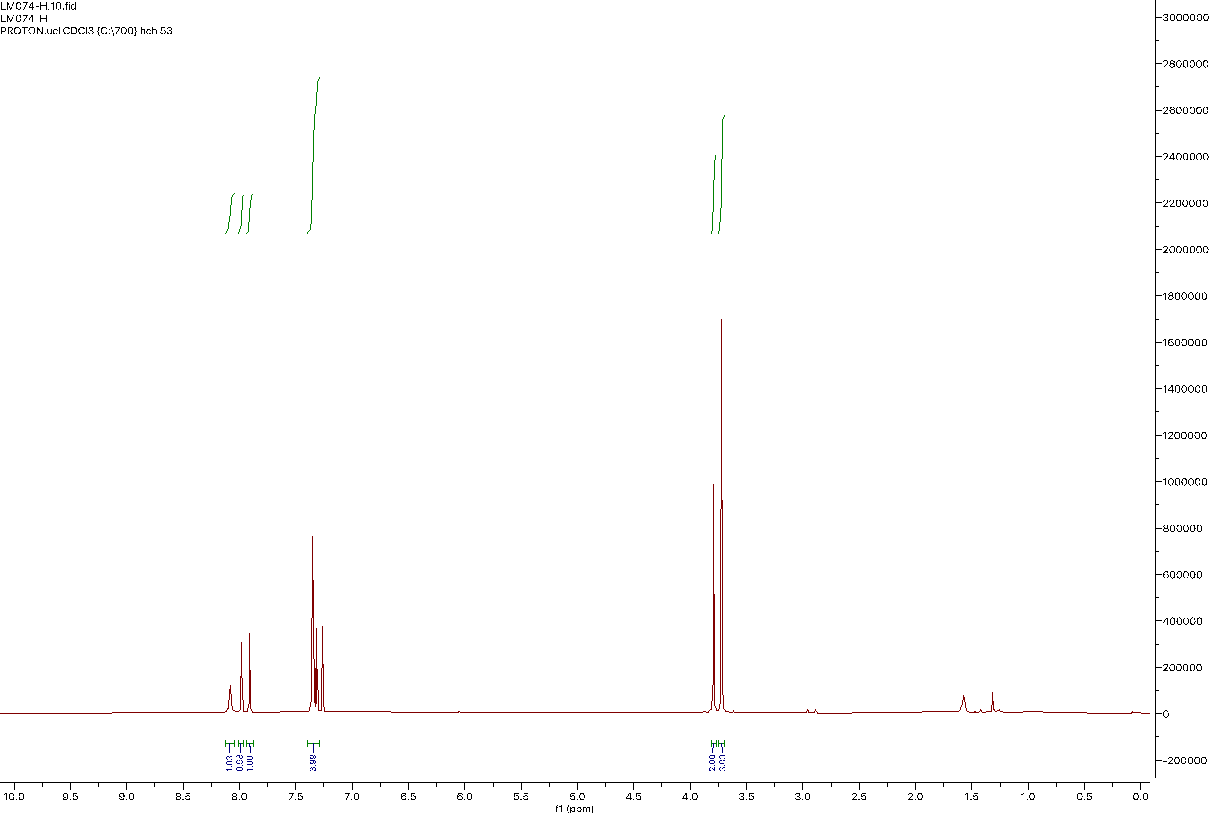


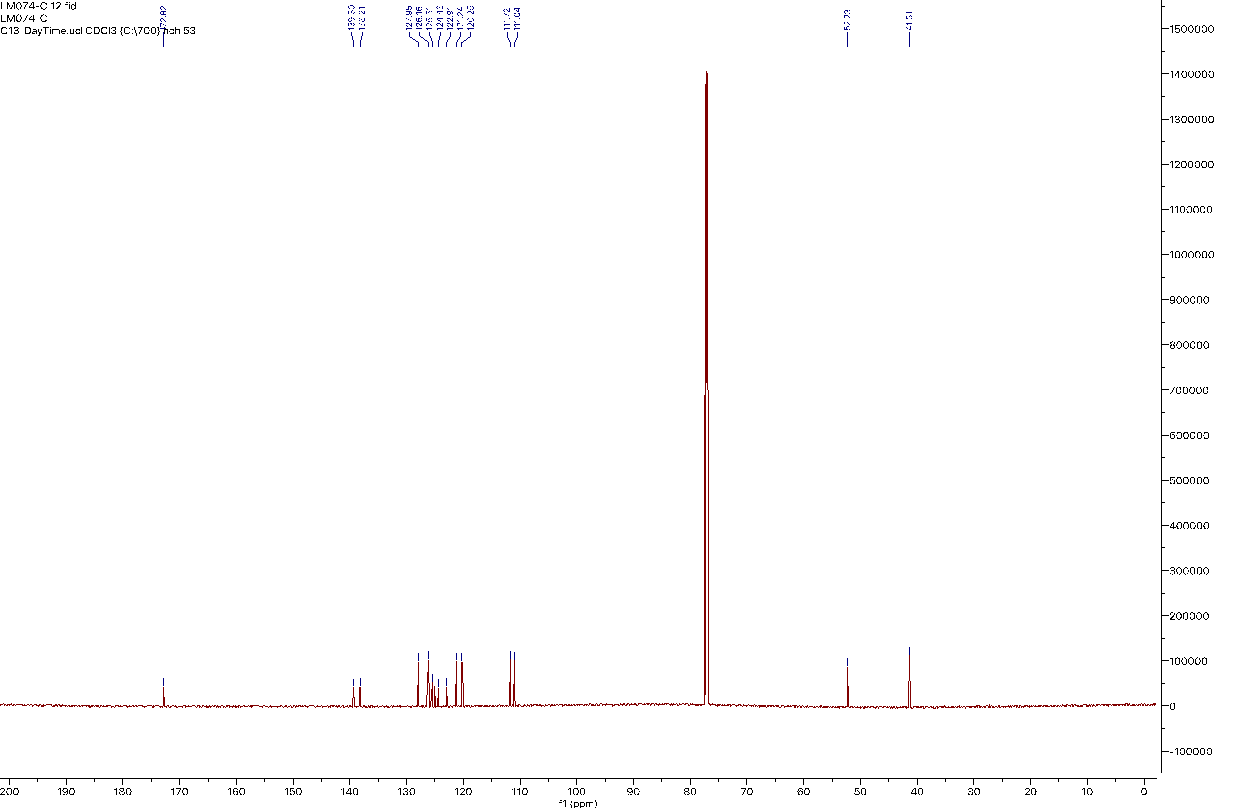


##### **2-(6-Chloro-9*H*-carbazol-3-yl)acetic acid 2**

To a solution of methyl 2-(6-chloro-9H-carbazol-3-yl)acetate (35 mg, 0.13 mmol) in a mixture of dichloromethane (4 ml) and methanol (0.5 ml) was added a methanolic solution of NaOH (2 M, 2 mL, 0.94 mmol). The reaction mixture was stirred for 20 h at RT. The solvent was then removed under reduced pressure. The crude product was added to distilled water and washed with diethyl ether (3 x 20 mL). The aqueous layer was then acidified by the addition of HCl_(aq)_ (1 M) and extracted with diethyl ether (3 x 20 mL). The organic layer was dried (Na_2_SO_4_) and the solvent was removed under reduced pressure. The crude material was recrystallised (diethyl ether and pentane) to afford the product as a crystalline solid (10.6 mg, 32%). M.p. slow decomposition 215–232 ^o^C (diethyl ether/pentane); *ν*_max_/cm^-1^ (neat) 3404, 3347, 3020, 2948, 2921, 1693; ^1^H NMR (700 MHz; CD_3_OD) δ 8.01 (1H, d, *J* = 2.1 Hz, 5-**H**), 7.94 (1H, br s, 4-**H**), 7.35­–7.40 (2H, m, 1-**H**, 8-**H**), 7.33 (1H, dd, *J* = 8.3, 1.7 Hz, 2-**H**), 7.31 (1H, dd, *J* = 8.5, 2.1 Hz, 7-**H**), 3.74 (2H, s, C**H**_2_); ^13^C NMR (176 MHz; CD_3_OD) δ 176.4, 141.0, 140.0, 128.7, 126.6, 126.4, 125.3, 125.0, 123.5, 121.8, 120.5, 112.8, 111.8, 41.9; *m/z* [ES+] 262 ([^37^Cl][MH]^+^, 10%), 260 ([^35^Cl][MH]^+^, 45%), 216 ([^37^Cl] [M–COOH]^+^, 25%), 214 ([^35^Cl][M–COOH]^+^, 75%); *m/z* [HRMS, ES+] found [M]^+^ 259.0398. C_14_H_10_^35^ClNO_2_ requires 259.0400.


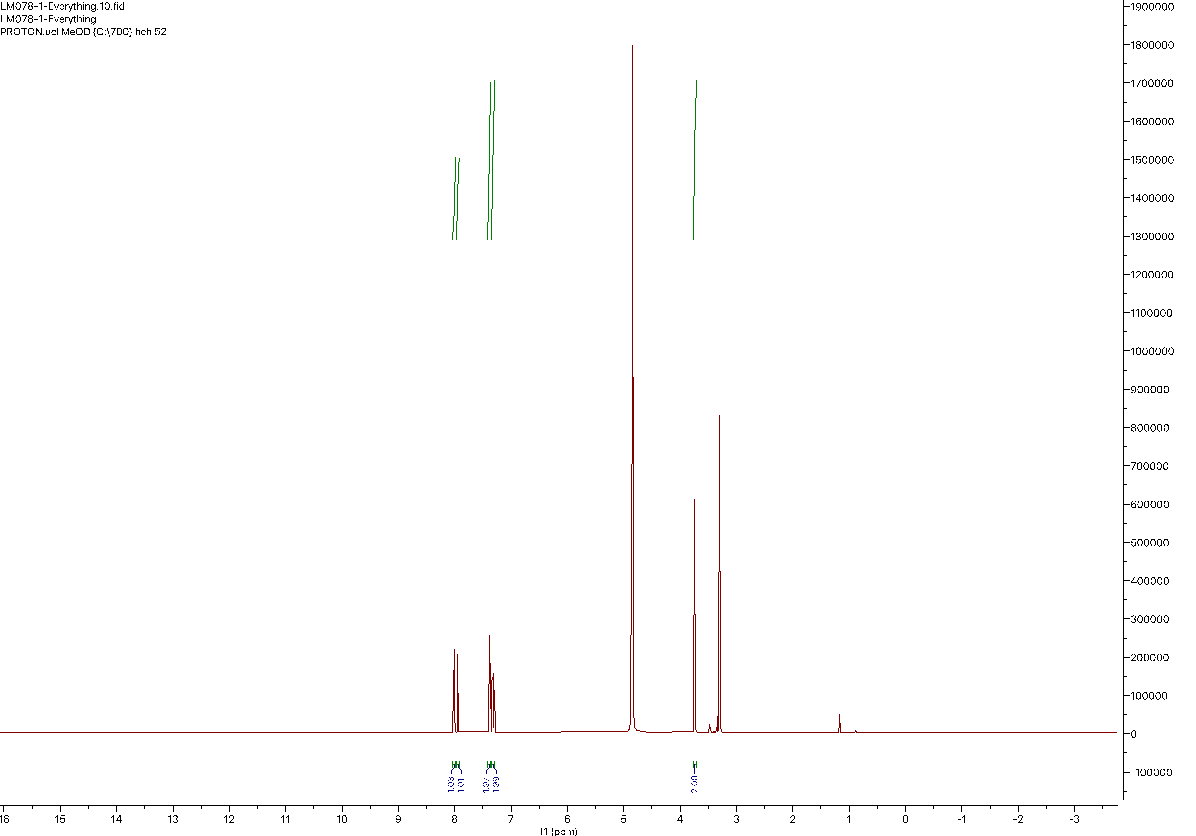


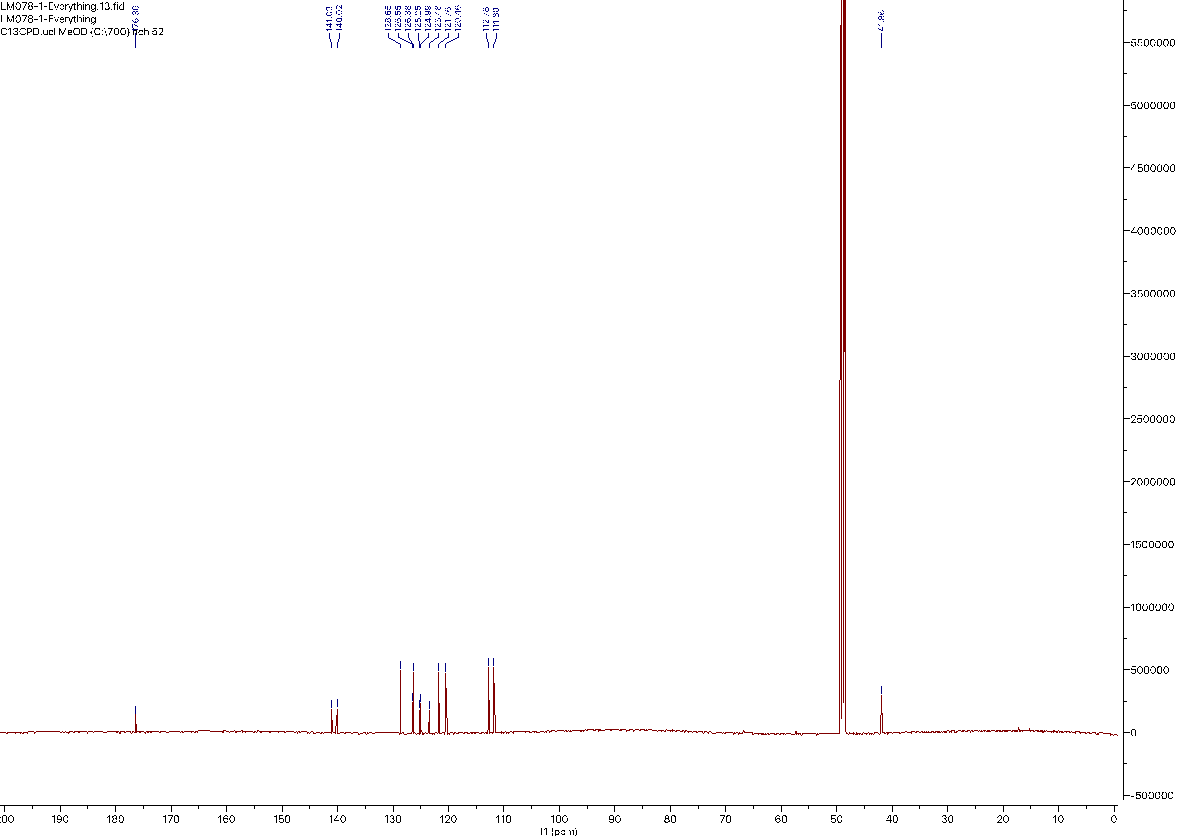

Supplement: dkaa307_supplementary_data [file dkaa307_supplementary_data.zip › Supplementary_Data_I.docx]
